# Comparison of the gut virus communities between patients with Crohn’s disease and healthy individuals

**DOI:** 10.3389/fmicb.2023.1190172

**Published:** 2023-06-15

**Authors:** Yuzhu Ding, Mengtian Wan, Zheng Li, Xiao Ma, Wen Zhang, Min Xu

**Affiliations:** ^1^Department of Gastroenterology, Affiliated Hospital of Jiangsu University, Jiangsu University, Zhenjiang, Jiangsu, China; ^2^Qinghai Institute for Endemic Disease Prevention and Control, Xining, Qinghai, China; ^3^Department of Laboratory Medicine, School of Medicine, Jiangsu University, Zhenjiang, Jiangsu, China

**Keywords:** inflammatory bowel disease, Crohn’s disease, viral metagenomics method, polyomavirus, anellovirus, CRESS-DNA virus

## Abstract

**Introduction:**

The escalating incidence of Crohn’s disease (CD), a debilitating ailment that ravages individuals and their families, has become a formidable issue over recent decades.

**Method:**

In this study, fecal samples from patients with CD and healthy individuals were investigated by means of viral metagenomics.

**Results:**

The fecal virome was analyzed and some suspected disease-causing viruses were described. A polyomavirus named HuPyV with 5,120 base pairs (bp) was found in the disease group. In a preliminary analysis employing large T region-specific primers, it was found that HuPyV was present in 3.2% (1/31) of healthy samples and 43.2% (16/37) of disease samples. Additionally, two other viruses from the anellovirus and CRESS-DNA virus families were found in fecal samples from CD patients. The complete genome sequences of these two viruses were described respectively, and the phylogenetic trees have been built using the anticipated amino acid sequences of the viral proteins.

**Discussion:**

Further research is required to elucidate the relationship between these viruses and the onset and development of Crohn’s disease.

## 1. Introduction

The intriguing roles played by gut microbiota in shaping human health encompass multifarious facets, including but not limited to the maturation and education of host immune responses, protection against the proliferation of enteric pathogens, and response to or modification of specific drugs (Schirmer et al., 2019). Consequently, several prevalent clinical maladies, such as the enigmatic inflammatory bowel disease (Nishida et al., 2018), the insidious atherosclerosis (Jonsson and Bäckhed, 2017), and the vexing asthma (Barcik et al., 2020), have been unequivocally linked to the fickle and enigmatic landscape of the gut microbiome.

Inflammatory bowel diseases (IBD) including Crohn’s disease (CD) and ulcerative colitis (UC), represents a chronic and non-specific inflammatory disease of the intestine whose etiology has yet to be fully explicated. The affliction, which often rears its head during childhood, adolescence, or young adulthood, poses a common occurrence in the clinical milieu (Ramos and Papadakis, 2019). Despite its ubiquity, the distribution of IBD is largely skewed toward Western countries, especially North America and Europe. Previous studies have indicated that the incidence of IBD is intimately linked to the twin forces of industrialization and urbanization, both of which continue to facilitate its spread and proliferation (Kaplan and Ng, 2017). Practically all industrialized countries are witnessing a surge in the number of patients grappling with IBD, with China serving as a classic example (Flynn and Eisenstein, 2019). Although the first IBD patient in China was only discovered as recently as 1956, the past two decades have witnessed an unyielding upsurge in the prevalence of IBD in the Asian giant (Wang et al., 2010). In terms of clinical symptoms, the prototypical manifestation of IBD comprises abdominal pain, fever, and diarrhea, among others. In its more pernicious forms, IBD can even progress toward carcinogenesis. The severity of symptoms is linked to the degree and extent of inflammation (Flynn and Eisenstein, 2019).

While the pathogenesis of IBD has not been identified clearly, there is growing consensus within the scientific community that the gut bacteriome, a vast and intricate network of microorganisms residing within the gastrointestinal tract, plays a pivotal role in its development and progression. One emerging theory posits that a reduction in the diversity of gut microbes can trigger IBD flares, disrupting the finely-tuned equilibrium of the gut ecosystem and unleashing a cascade of pathological events (Ott et al., 2008). However, the preponderance of studies conducted on the gut microbes of IBD patients still tilts toward the bacterial dimension, relegating the viral aspect to the periphery. In fact, the gut virome exerts its influence through intricate interactions with the gut bacterial community (Imai et al., 2022). Moreover, the viral genome of gut microbes remains far from being fully explored, with a panoply of uncharted viruses, boasting high genetic and morphological diversity, lurking in the shadows and eluding the grasp of scientific inquiry (Guo et al., 2017). In fact, Tomofuji et al. (2022) found that the *Podoviridae* family exhibited a significant reduction in autoimmune disease patients’ intestines, underscoring the pivotal role of the viral dimension in shaping the trajectory of the disease. Zuo and colleagues have observed the enrichment of *Caudovirales* phage in the UC mucosa (Zuo et al., 2019). Norman et al. (2015) also noting a distinct increase in the richness of the *Caudovirales* phage in the gut of patients with IBD. Thus, the viral genome of gut microbes cannot be overlooked in the progression of the disease.

The scintillating advent of metagenomic next-generation sequencing has been nothing short of a revolution, heralding a new era in environmental analysis. The phenomenal success of this trailblazing technology has rapidly catapulted it to the forefront of medical and clinical practice, with the untold potential of unlocking a treasure trove of insights into the enigmatic workings of the human gut. In this study, a total of 37 fecal samples harvested from Crohn’s disease patients and 9 fecal samples from healthy individuals, were subjected to analysis using this innovative technology, with a focus on scrutinizing the enteric virome in the quest to identify any lurking enteroviruses with putative links to Crohn’s disease. In particular, members of the *Polyomaviridae*, *Anelloviridae* and CRESS-DNA virus were scrutinized with the utmost scrutiny, in the hope of unraveling the elusive and enigmatic pathogenesis of Crohn’s disease.

## 2. Materials and methods

### 2.1. Sampling

From the Affiliated Hospital of Jiangsu University of Jiangsu, China, 37 fecal samples were gathered from Crohn’s disease (CD) patients who had received a diagnosis, alongside 9 fecal samples from healthy individuals at the Jingkou Community Hospital of Jiangsu, China, who had been excluded from the group of IBD by gastroscopy. The study specified certain criteria for sample selection, including no use of probiotics within 3 weeks, antibiotics, or antimicrobial agents, no intestinal tumors or other diseases of the digestive system, and residency in Zhenjiang, Jiangsu of China. To avoid the effects of diarrhea on enteroviruses, all disease samples were taken from patients in clinical remission. And all patients received standard treatment with infliximab every 2 months. Furthermore, all participants were required to be between the ages of 20–50 years old, and all individuals signed a written consent form prior to participation in the study. The demographic data and clinical characteristic of the patients included in this study were shown in Supplementary Table S3.

### 2.2. Viral metagenomic analyses

Each frozen fecal sample was thawed at ambient temperature environment and mixed with phosphate-buffered saline (PBS) solution. After vigorous vortexing, the mixture was subjected to a high-speed centrifugation at 14,000 × *g* for 10 min to remove the bacteria and large particular matter roughly. The resulting supernatant was filtered through a 0.45 um filter to exclude eukaryotic and bacterial cell-sized particles. The filtrate, enriched in viral particles, was treated with a mixture of DNases and RNase to digest unprotected nucleic acid (Yang et al., 2021). Total nucleic acid was then extracted from the remaining viral particles using the QIAamp Viral RNA mini Kit (QIAGEN). To prepare the samples for sequencing, independent libraries were created using the Nextera XT DNA Sample Preparation Kit (Illumina), and the libraries were sequenced using the Illumina MiSeq platform with 250 bp paired-end reads with dual barcoding for each pool.

### 2.3. Bioinformatics analysis

The Illumina Miseq platform was used to generate paired-end reads of 250 bp, which were decoded using vendor software. In the present study, an in-house analysis pipeline running on a 32-node Linux cluster was used to treat the data. To improve data quality, clonal reads were removed and low-quality sequencing tails were trimmed using Phred, with a quality score threshold of ten. Adaptors were trimmed using the default parameters in VecScreen, which uses NCBI BLASTn with specialized parameters designed for adapter removal. The cleaned reads were then *de novo* assembled using SOAPdenovo2 version r240, with a Kmer size of 63 and default settings. The assembled contigs and singlets were compared to an in-house viral proteome database using BLASTx, with an *E*-value cutoff of <10^−5^.

### 2.4. Genome sequencing and PCR screening

In order to get a preliminary understanding of the prevalence of polyomavirus, anellovirus and CRESS-DNA virus in patients with Crohn’s disease, a nested PCR approach was employed. In addition to the samples used to construct the libraries, 22 additional samples collected from healthy individuals at Jingkou Community Hospital of Jiangsu were also included in the study. The criteria for sample selection were the same as previous 9 samples used for constructing libraries.

To obtain the complete genome sequence of a novel anellovirus and CRESS-DNA virus, inverse PCR and Sanger sequencing were carried out. The primers used in there PCR steps were based on contigs of virus that were assembled from the sequence reads in the libraries. The specific sequences for each primer used in the PCR are shown in Tables 1, 2.

**Table 1:** Nested PCR primers designed for screening the positive samples.

Virus	Primer	Application	Primer sequence (5′-3′)
Polyomavirus	polyoWF	First round	AGCAGCCAAACAAAGGGTTG
	polyoWR		GTTGCTTATGCCATGCCCTG
	polyoNF	Second round	GGGTGGCCTGGATTCATTGT
	polyoNR		ATCCCTTGACTCTGCACCAG
Anellovirus	anelloWF	First round	AACCAGCCACAACTCCACAA
	anelloWR		TCGTTAGTGGTGAGCCGAAC
	anelloNF	Second round	GGGGACTGTGCACCACTAAA
	anelloNR		GGCTGTCATGTCAGTGTCGT
CRESS-DNA virus	CRESSWF	First round	AAACCGCCCTTGGACTATCG
	CRESSWR		CGCTCGAACCCTCGTTGTAT
	CRESSNF	Second round	TAATCAGGCGGTGCGTAAGG
	CRESSNR		GGTTGTTGGCATCTACAGCG

**Table 2:** Inverse primers used to generate the complete genome sequences of the novel anellovirus and CRESS-DNA virus.

Virus	Primer	Application	Primer sequence(5′-3′)
Anellovirus	TTV-WF	First round	TCTCCAACTCGCCCAAGTTC
	TTV-WR		ACTGCCTCTCAATTCCACGG
	TTV-NF	Second round	ACCTCTACTCCCTCCAGCAG
	TTV-NR		TGGAGTATGCACCAGGGACA
CRESS-DNA virus	Circo-WF	First round	AACGCCTCCAACTGACCAAT
	Circo-WR		CGATAGTCCAAGGGCGGTTT
	Circo-NF	Second round	CGCTGTAGATGCCAACAACC
	Circo-NR		TAGTCCAGTACGCAGCCTCT

### 2.5. Phylogenetic analyses

The phylogenetic analyses were conducted using either the complete genome or partial gene sequences. The sequence alignment was performed by CLUSTAL W with the default settings. The phylogenetic trees were constructed using MrBayes3.2.7. The Markov chain was run for a maximum of 1 million generations, sampled every 50 generations, and the first 25% of Markov chain Monte Carlo samples were discarded as burn-in. Maximum-likelihood trees were also constructed to confirm all the Bayesian inference trees, using MEGA software version 10.1.8 (Lu et al., 2021).

## 3. Results

### 3.1. Viral metagenomic overview

**Figure 1:**
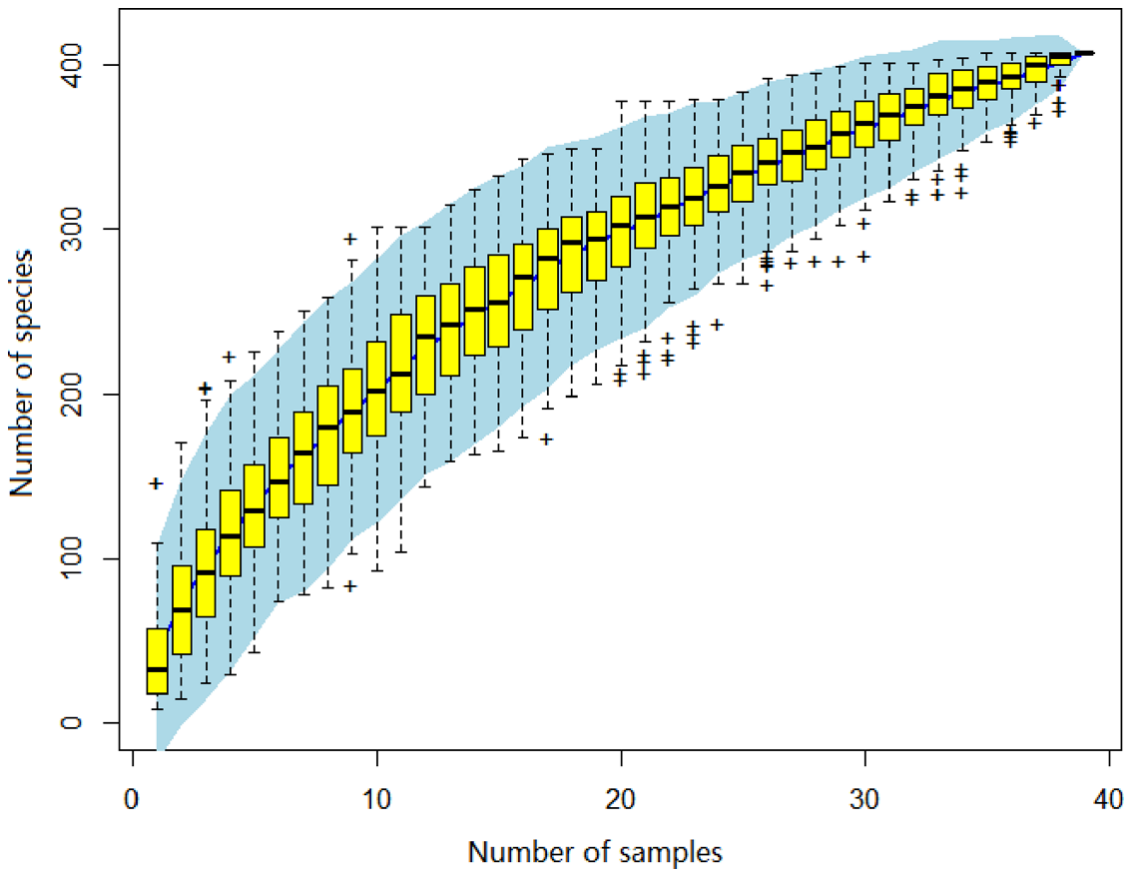
The virus species accumulation curve, with its abscissa denoting the number of libraries, and the ordinate represents the number of species identified. The blue shading visible on the plot depicts the 95% confidence interval.

**Figure 2:**
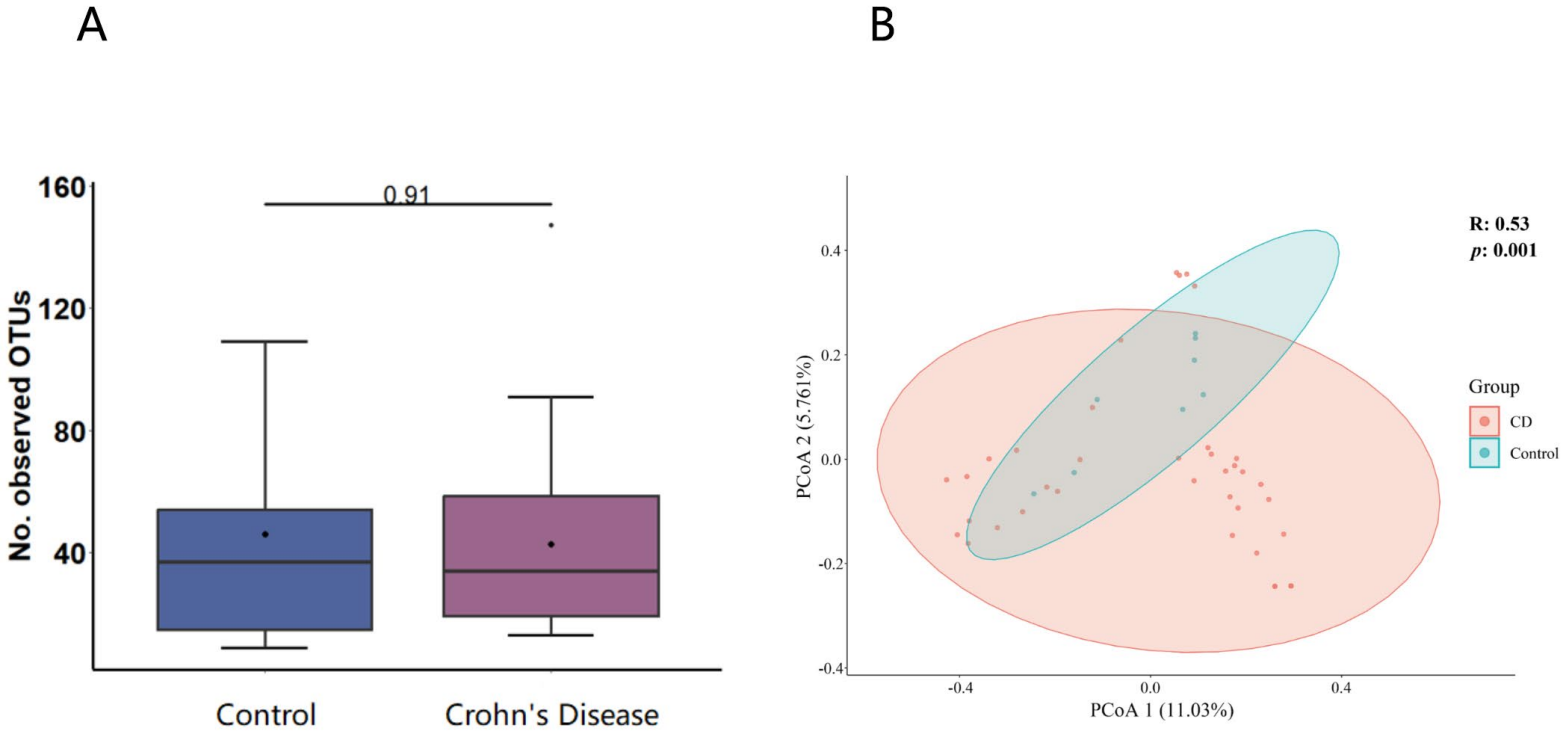
**(A)** α-diversity (virome composition comparison of the CD patients to healthy individuals). The horizontal bars contained within the boxes on the graph represent medians. The tops and bottoms of boxes indicate the 75th and 25th percentiles, respectively. **(B)** *β*-diversity analysis (Principal coordinate analysis scatter plot). The circles in the plot represent the 95% normal probability ellipse for each group, with red corresponding to the Crohn’s disease (CD) group, and blue corresponding to the control group.

**Figure 3:**
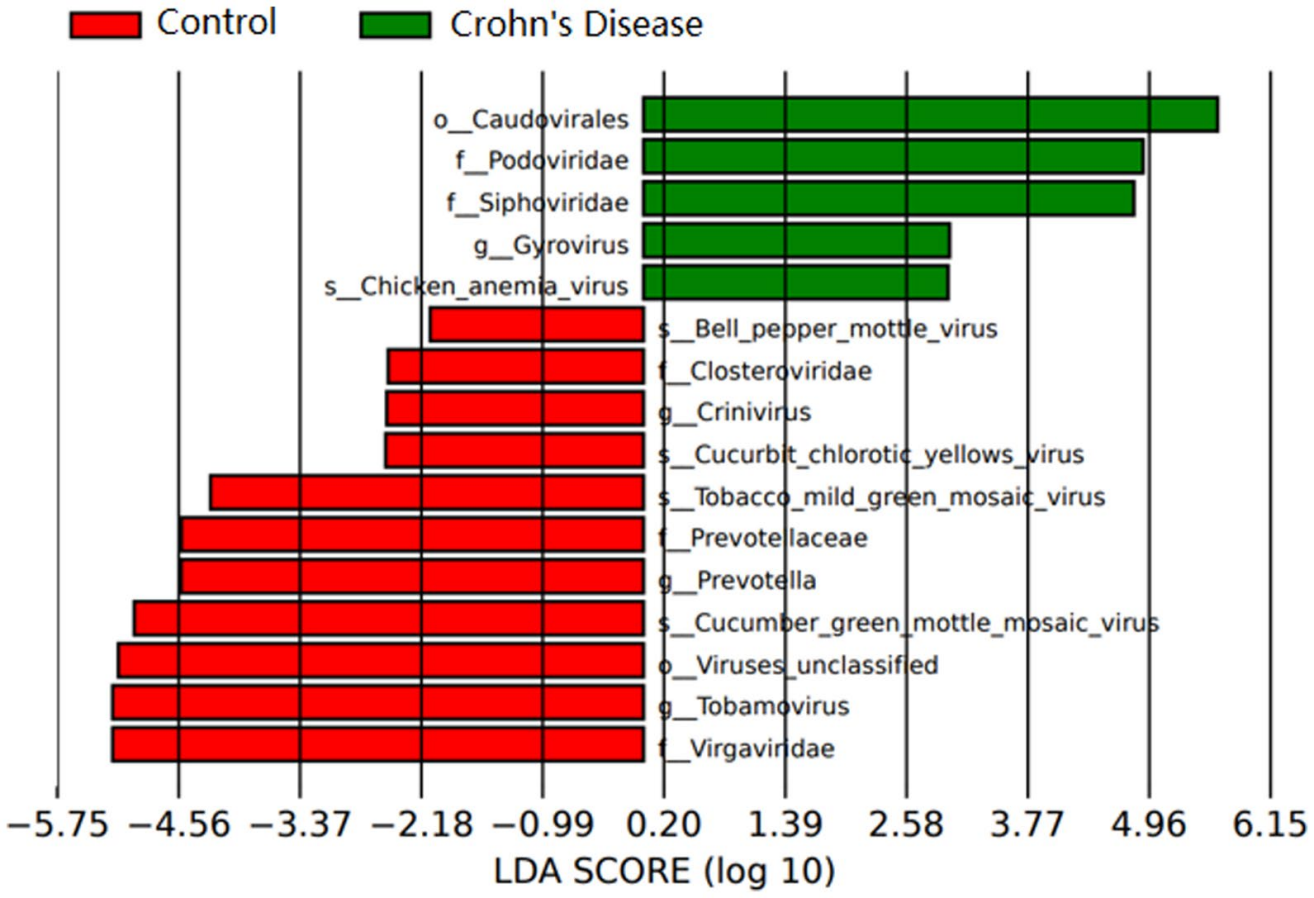
Relative abundance of virus groups according to LEfSe in CD patients and healthy individuals. Only taxa with LDA values of 2.0 or higher are shown.

**Figure 4:**
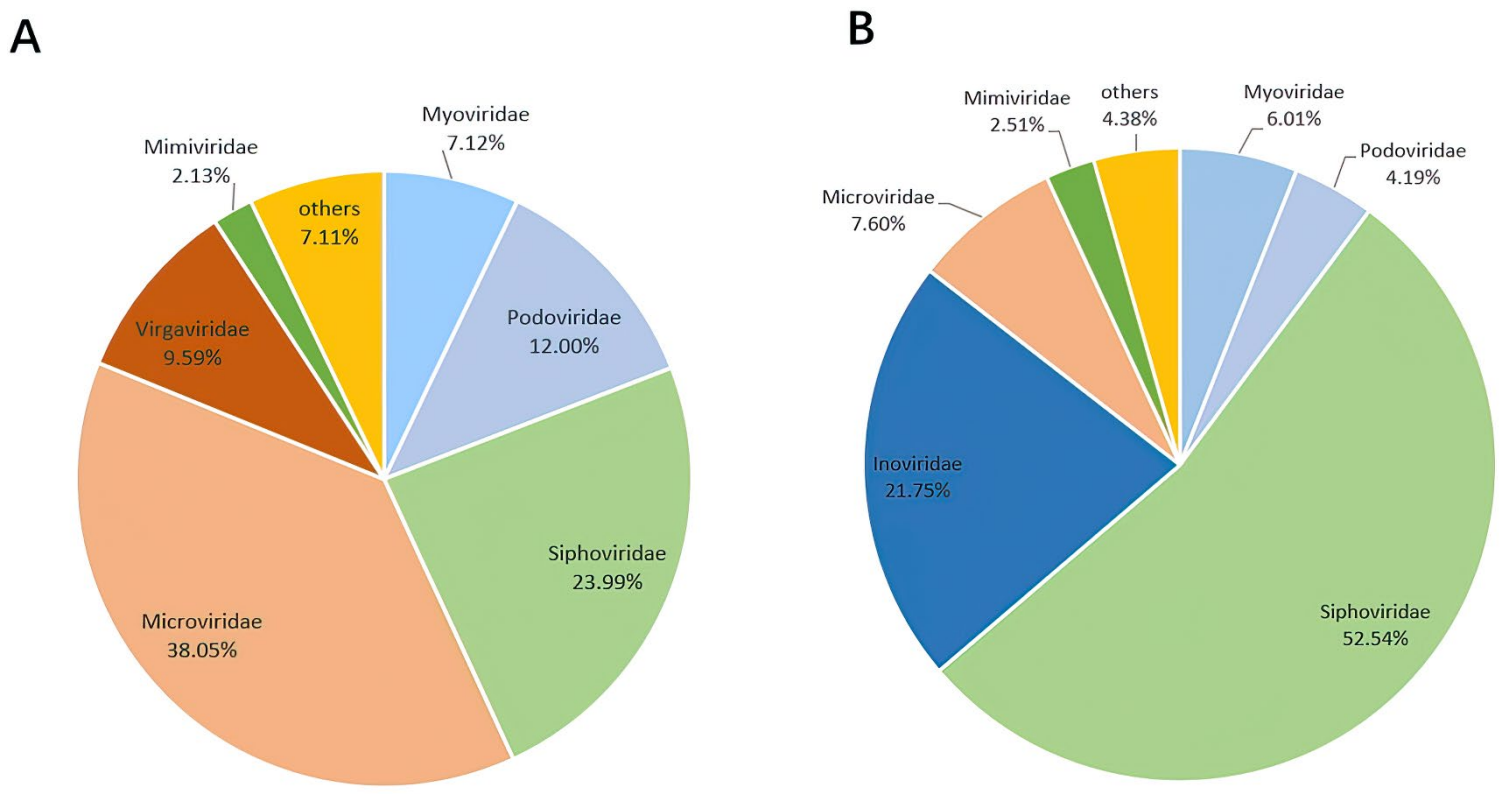
The composition of the virus community. **(A)** Disease group. **(B)** Healthy group. The figure show the viruses with a proportion of more than 2%.

**Figure 5:**
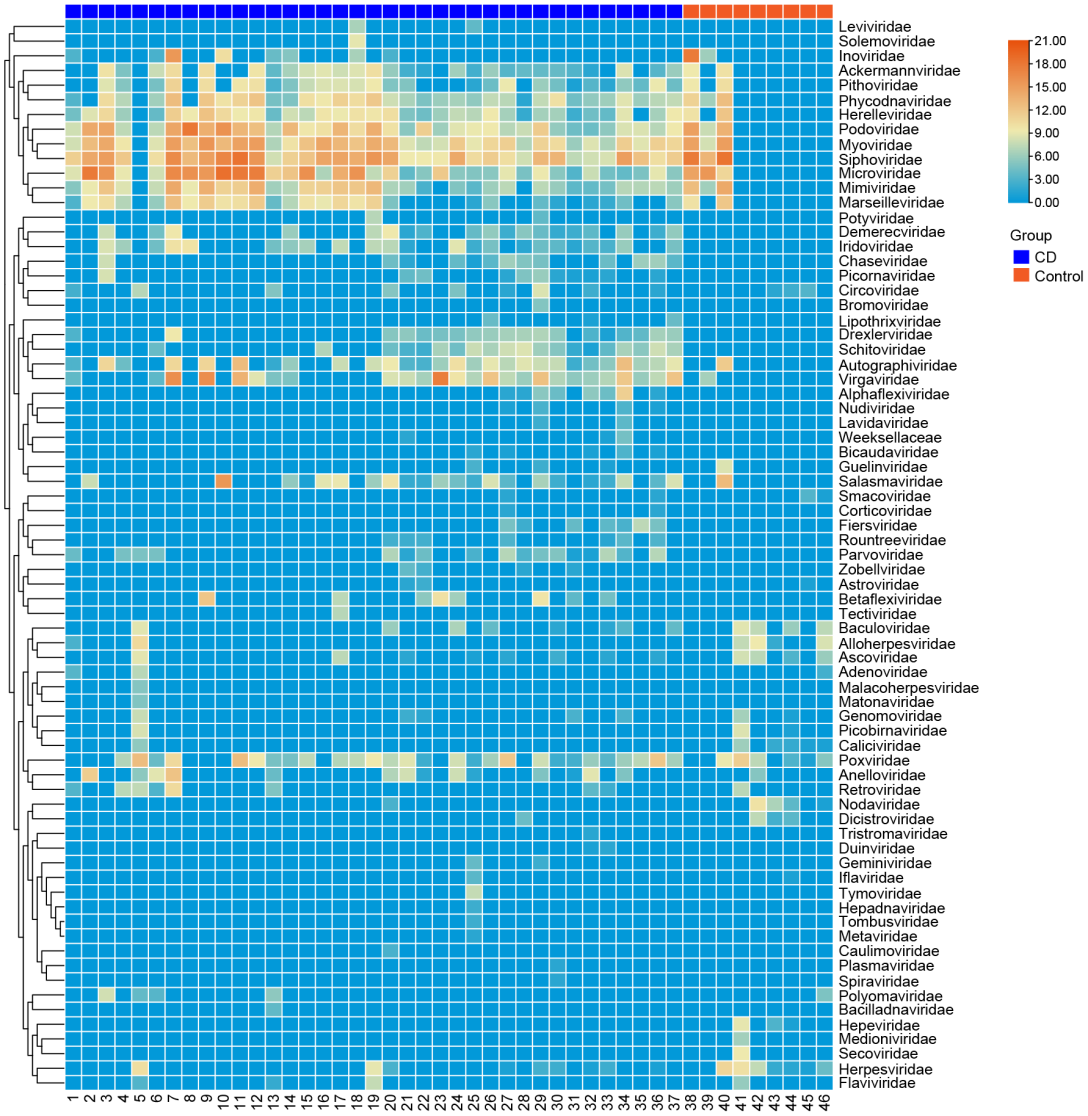
Clustering heatmap of representative virus families from 46 libraries. The number of libraries is indicated at the bottom of the figure. The blue bar at the top represents the disease group, and the red bar represents the healthy group. The name of the virus is shown on the rights side of the figure. The number of reads is presented in log10 scale.

A total of 3,985,095 unique sequence reads were obtained from the 46 libraries, as shown in Supplementary Tables S1, S2. By plotting a Species Accumulation Curves, we were able to obtain a clear picture of the richness of the virome (Figure 1). As the number of samplings increases, the curve gradually transitioned to a plateau, indicating that the sample size in this study was sufficiently large and that additional data would only reveal a small number of new species. There was no significant difference in virome richness between the disease and control groups (Wilcoxon test, *p* = 0.91; Figure 2A). Nevertheless, in terms of beta diversity, the principal coordinate analysis (PCoA) indicates that there is a certain degree of difference in the viral composition between the two cohorts (Figure 2B). In particular, LEfSe analysis revealed that patients with Crohn’s disease had higher LDA scores in *Caudovirales*, *Podoviridae*, *Siphoviridae*, *Gyrovirus*, *Chicken anemia virus*, while the control group demonstrated higher LDA scores in *Virgaviridae*, *Bell oeoper mottle virus*, *closterovirodae*, *Crinivirus*, *Cucurbit chlorotic yellows virus*, *Tobacco mild green mosaic virus*, and etc. (Figure 3). This means these features with high LDA scores are more likely to be responsible for the observed differences between the two cohorts compared. The viral composition from different families in the disease and control groups was analyzed. The pie chart showed that the enteric virome of patients with CD was dominated by *Microviridae* (38.05%), and *Siphoviridae* (23.99%), followed by *Podoviridae* (12.00%), *Virgaviridae* (9.59%), *Myoviridae* (7.12%), *Mimiviridae* (2.13%; Figure 4A). In contrast, the most abundant virus families in the control group were *Siphoviridae* (52.54%), *Inoviridae* (21.75%), *Microviridae* (7.60%), *Myoviridae* (6.01%), *Podoviridae* (4.19%), *Mimiviridae* (2.51%; Figure 4B). The viral family composition of the different libraries in the disease and control groups were plotted separately as heat maps (Figure 5). Due to the large size of the gut virus family, we cannot study all families in the paper. Therefore, in this study, we chose the following three families that we were interested in and conducted a detailed investigation. The issue of Polyomaviruses and their potential impact on individuals with Crohn’s disease has been a topic of interest in previous published studies, wherein it was observed that such viruses were detected at higher rates in the intestinal tissues of patients with Crohn’s disease when compared to healthy individuals (Miskin and Koralnik, 2015). Moreover, there have also been several previous studies that examined the seroprevalence of Polyomaviruses in children with IBD (Hradsky et al., 2015). Interestingly, anelloviruses have also been reported to be more prevalent in patients with inflammatory bowel disease compared to their healthy counterparts (Liang et al., 2020). Given the ubiquitous presence of CRESS DNA viruses in the natural world and the vast array of unclassified viruses they contain, our study has taken the initiative to investigate their potential role in Crohn’s disease pathogenesis. It is our hope that this study will provide valuable insights into the complex interplay between viruses and Crohn’s disease.

### 3.2. Polyomavirus

Polyomaviruses (PyVs) are small non-enveloped viruses with a circular double-stranded DNA genome (Oliver-Guimerá et al., 2021). The genome is approximately 5,000 bp and is composed of 88% protein and 12% DNA (Eash et al., 2006). The genome mainly encodes five major structural proteins, which are responsible for its unique features. These proteins include large tumor antigen (LTAg), small tumor antigen (STAg), the viral capsid proteins VP1, VP2, and VP3. Additionally, some specific viruses also encode a multifunctional nonstructural protein called agnoprotein (Boothpur and Brennan, 2010).

Polyomavirus, initially known as murine K virus, was first discovered in Kilham (1952). It was found to cause tumors, such as adenocarcinoma, in mice upon infection (Gross, 1953). The two most common PyVs that infect humans are JC virus and BK virus. Some research has associated BK virus with kidney involvement, such as BK virus-associated nephropathy (BKVAN; Pinto and Dobson, 2014), while the JC virus has been proved contribute to tumorigenesis, including neurological and gastrointestinal lesions, such as gliomas, ependymomas, and colorectal cancer (Padgett et al., 1971; Burnett-Hartman et al., 2008). As research has progressed, four other human polymaviruses have been identified, including WU polyma virus, KI polyma virus, Merkel cell polyoma virus, and trichodysplasia spinulosa virus (Pinto and Dobson, 2014). However, the exact relationship between these four viruses and human diseases is still unclear.

**Figure 6:**
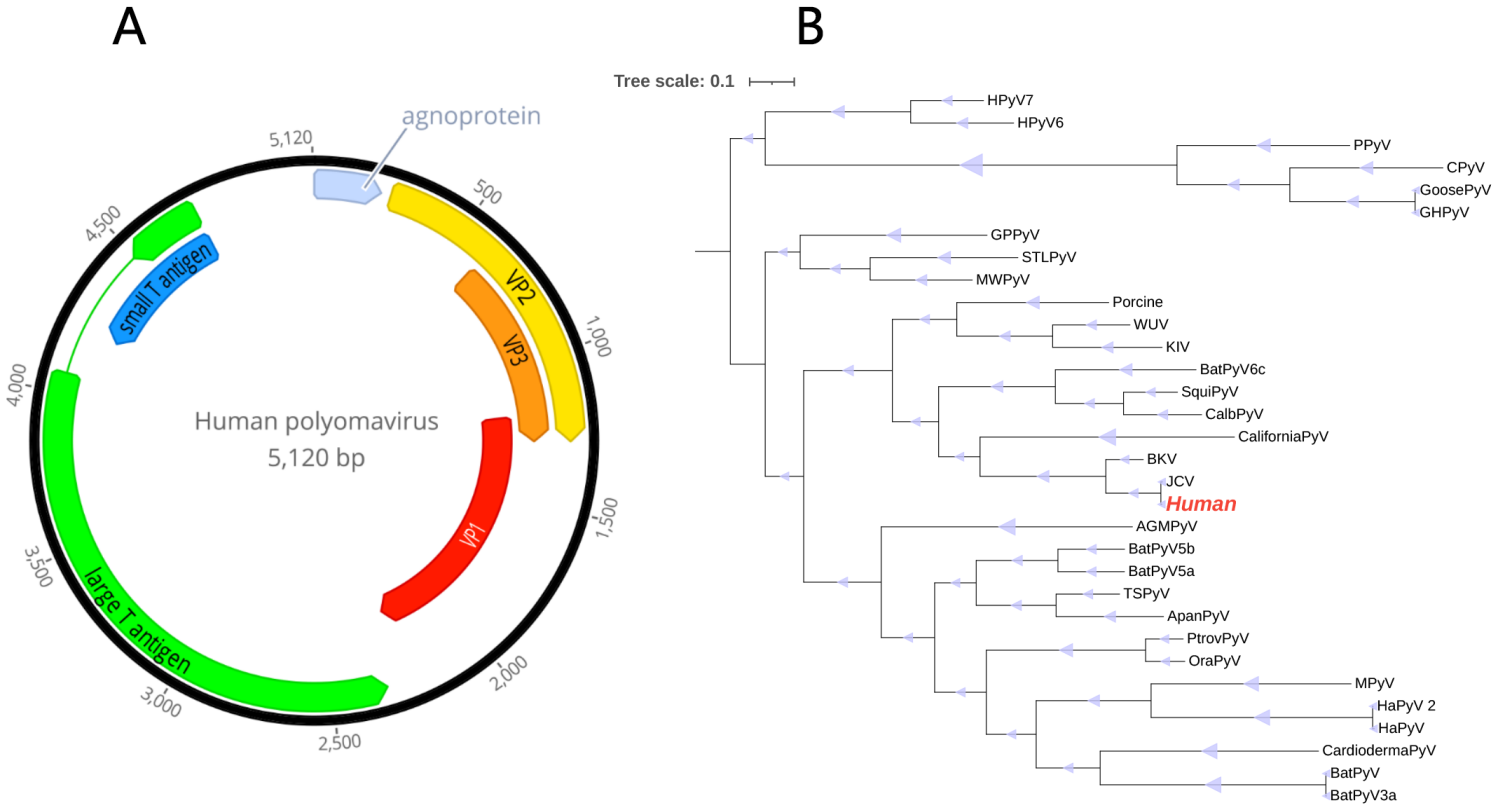
**(A)** Genome organization of the human polyomavirus, detected in fecal sample of a patient with Crohn’s disease. **(B)** Phylogenetic tree based on the amino acid sequences of the LTAg protein. The virus found in this study are marked with red.

In this investigation, a fecal sample pool (human03) obtained from a Crohn’s disease patient was analyzed, and a human polyomavirus (HuPyV) were identified. The full-length genome of this HuPyV comprised 5,120 bp and had an overall GC content of 40.3%. Like other members of the polyomavirus family, it was found to code for five major proteins, including the large T antigen (LTAg) which was made up of 661 amino acids, the small T antigen (STAg) composed of 173 amino acids, and VP1, VP2, and VP3 with 357,345, and 226 amino acids, respectively. Furthermore, an agnoprotein was predicted to be present (Figure 6A; Yamaguchi, 1992).

PCR screening was performed on a total of 68 samples collected, including 46 samples used for library construction and an additional 22 samples collected from healthy individuals. In total, 37 disease samples and 31 control samples were taken. PCR screening was carried out on these samples, and the results reveals that 16 out of 37 (43.2%) samples in the disease group were positive, while only 1 out of 31 (3.2%) were positive in the control group. Subsequently, the amplified fragments were sequenced using the Sanger method, and the primers that were employed in the PCR reaction were designed based on the LTAg.

With an aim to ascertain the genetic relationship between HuPyV and the other Polyomavirus species, a phylogram was constructed using the LTAg, including HuPyV and other representative polyomavirus species. The results revealed that the HuPyV was significantly distinguished from other polyomaviruses, and instead clustered with JCPyV and BKPyV, forming a separate cluster (Figure 6B). Additionally, the complete genome of HuPyV displayed a maximum similarity of 99.67% with the JCPyV (AB103412) variant available on Genbank, indicating a high degree of resemblance between these two species.

### 3.3. Anelloviruses

Anelloviruses (AVs) are a group of circular single-stranded DNA viruses ranging from 2.1 to 3.9 kb in size. The most extensively researched member of this virus family in humans is the Torque teno virus (TTV), which was initially identified in the serum of a non-A-E hepatitis patient (Pisani et al., 2000). It belongs to the *Alphatorquevirus* genus. *Betatorquevirus* or Torque teno mini virus (TTMV), and *Gammatorquevirus* or Torque teno midi virus (TTMDV) are other viruses belonging to the Anellovirus group (Spandole et al., 2015). With the advent of metagenomics, many more novel anelloviruses have been identified, yet their impact on human beings remains largely elusive.

Bioinformatic analysis was executed and it revealed large numbers of anelloviral reads from these libraries. A long contig of 2,957 bp was assembled from one of the fecal pools, human02, which exhibited similarity to ORF1 of anellovirus. Additionally, PCR screening was performed on a total of 68 samples, including the 46 samples prepared for the libraries and 22 samples from healthy individuals. The results indicated that 5 out of the 37 (13.5%) samples in the disease group and 5 out of the 31 (16.1%) samples in the healthy group were positive.

**Figure 7:**
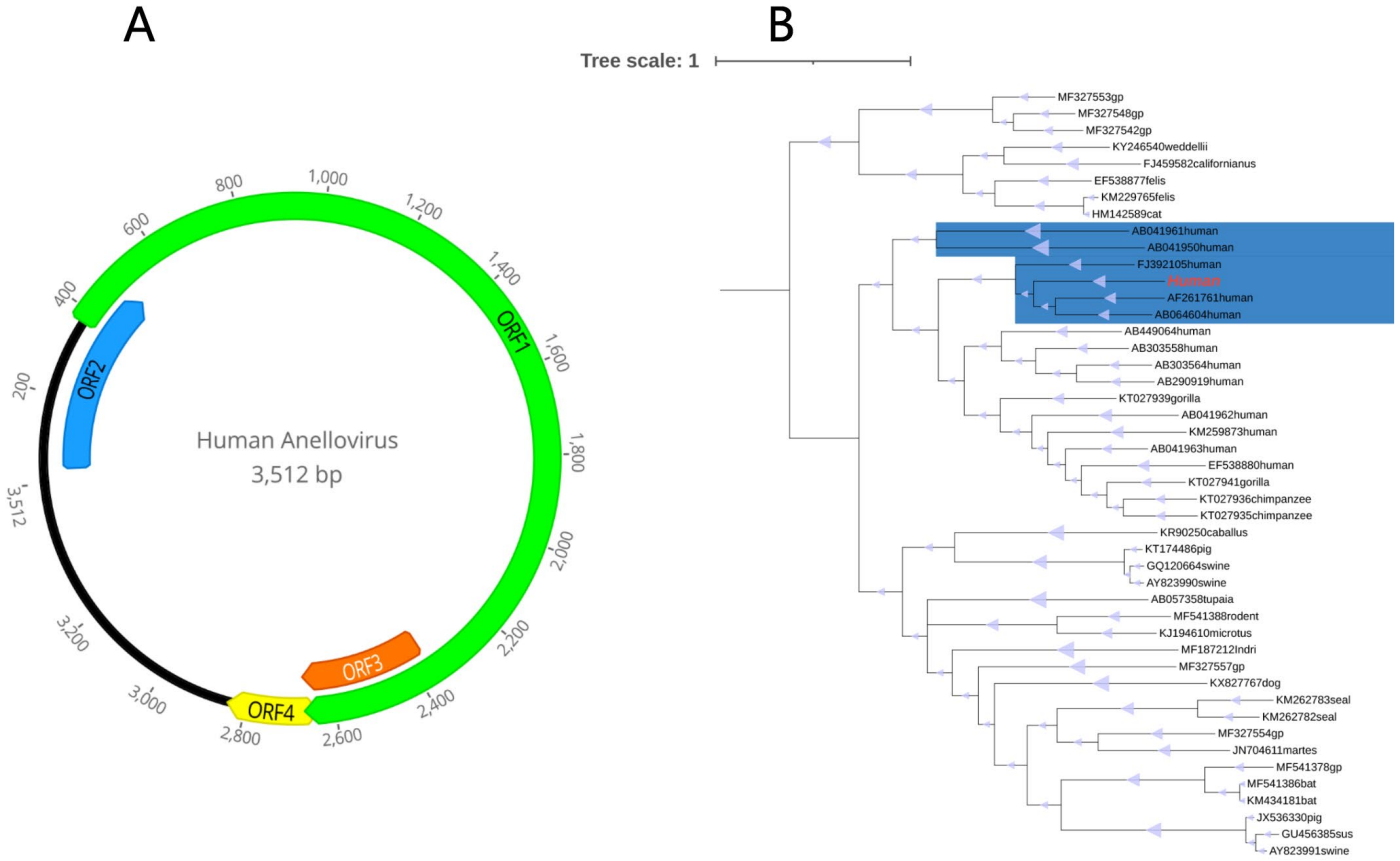
**(A)** Genome organization of the novel human anellovirus detected in fecal sample of a patient with Crohn’s disease. **(B)** Phylogenetic tree established based on the amino acid sequences of the ORF1 region. The virus found in this study are marked with red. The parts marked blue are *Alphatorquevirus*.

In an effort to gain a deeper understanding of HuAV, inverse PCR was utilized to determine the complete genome sequence. The resulting genome sequence was found to be 3,512 bp in length, with a G + C content of 51.2%. Notably, four main open reading frames (ORFs) were identified within the genome, including the largest ORF, ORF1, which encodes 765 amino acids. Additional ORFs were found to encode 156, 102, and 63 amino acids, respectively (Figure 7A).

Based on the ORF1 of the novel HuAV and a few representative members of the anellovirus family available in GenBank database, a phylogenetic tree was constructed, in order to determine the evolutionary relationship of this novel virus. Based on the results, this HuAV fell within the clade of the genus *Alphatorquevirus*, which was isolated from the serum of an acute non-A-E hepatitis patient (Genbank No.AF261761), the serum of TTV-infected infants (Genbank No. AB064604), and the serum of a healthy Taiwanese (Genbank No.FJ392105; Figure 7B).

### 3.4. CRESS DNA virus

Circular replication-associated protein (Rep)-encoding single-stranded DNA viruses (CRESS DNA viruses), encode replication initiator protein (Rep) and capsid protein (Cap). The International Committee on Taxonomy of Viruses (ICTV) has classified them into six families, including *Geminiviridae*, *Nanoviridae*, *Circoviridae*, *Genomoviridae*, *Bacilladnaviridae*, and *Smacoviridae*, which infect plants (Jeske, 2018), human (Tan et al., 2013), and certain animals (Li et al., 2013; Hansen et al., 2015; Fehér et al., 2017). Viral metagenomics has led to the detection of numerous novel and unclassified CRESS-DNA viruses in recent years, with a large proportion remaining unclassified (Guo et al., 2018). Abbas et al. (2019) identified a new family named *Redondoviridae* based on bronchoalveolar lavage samples.

This study uncovered numerous CRESS DNA virus reads from the library human08 and assembled a long 854 bp contig. Remarkably, this contig contains the sequence encoding the Capsid protein. Based on this sequence, inverse PCR and Sanger sequencing were performed, leading to the determination of the complete genome sequence of the virus.

**Figure 8:**
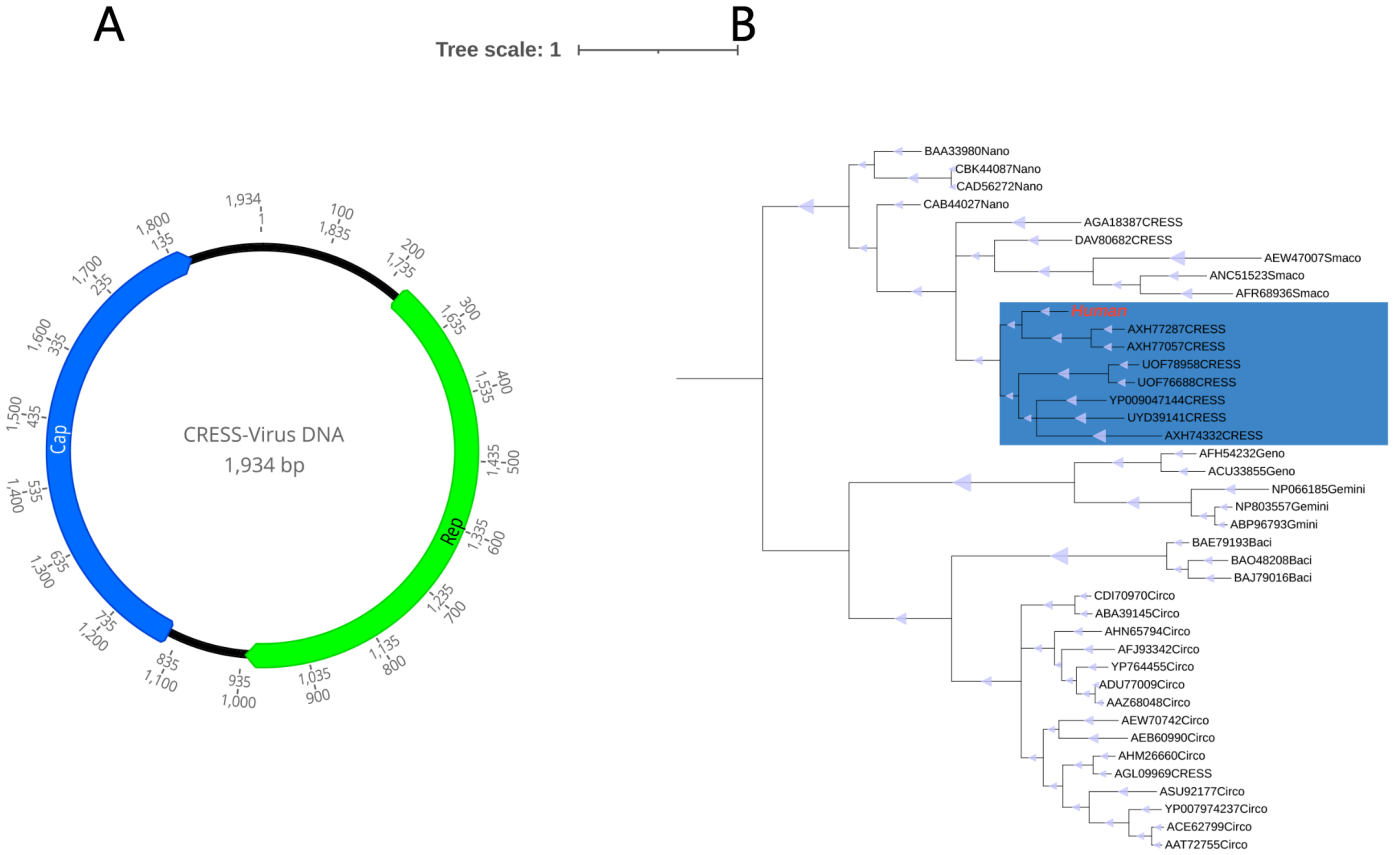
**(A)** Genomic organization of the novel human CRESS-DNA virus. **(B)** Phylogenetic tree based on the amino acid sequence of Rep protein. The virus found in this study are marked with red. The parts marked blue are unclassified CRESS-DNA virus.

In this investigation, an entirely new circular DNA virus, called HuCV, was discovered with a total genome length of 1934 bp and a GC content of 44%. The Rep protein (257 aa) and The Cap protein (237aa) were identified, respectively (Figure 8A). The results of PCR screening indicated that 6 out of the 37 (16.2%) samples in the disease group and 1 out of the 31 (3.2%) samples in the healthy group were positive.

For a better understanding of the evolutionary relationship between HuCV and other CRESS-DNA viruses, a phylogenetic analysis was carried out with HuCV, the Rep-representative viruses of the families *Geminiviridae*, *Genomoviridae*, *Bacilladnaviridae*, *Circoviridae*, *Smacoviridae,* and *Smacoviridae*, as well as a few unclassified CRESS-DNA viruses. It was found that this newly identified CRESS DNA virus is clustered in a separate branch with other two other unclassified CRESS-DNA virus strains isolated from abalone and haddock tissues (GenBank No. AXH77057.1 and GenBank No. AXH77287.1), with which it shared 44.40 and 43.24% amino acid sequence identity, This information is illustrated in Figure 8B.

## 4. Discussion

In this current investigation, the enteric virome was investigated in patients suffering from Crohn’s disease, as well as in healthy individuals. Despite observing no noteworthy differences in viral diversity between the disease group and the control group, the relative abundance of *Caudovirales* was higher in the CD group. Three viruses, including a polyomavirus, an anellovirus, and a novel CRESS-DNA virus, were investigated through metagenomic analysis in patients with CD and healthy controls. The positive rates of these viruses were evaluated and compared between the disease groups and control groups. These findings may offer valuable insights into the etiology studies of Crohn’s disease. The differences in viral taxa between patients with Crohn’s disease and healthy controls may have clinical significance, which suggests that enteroviruses may play a role in the development and progression of Crohn’s disease. This has important implications for the diagnosis and treatment of the disease, as it may provide new targets for therapy. Besides, the differences in viral taxa may have diagnostic implications. In the present study, specific viral taxa are found to be more prevalent in individuals with Crohn’s disease compared to healthy controls, this could potentially be used as a diagnostic marker for the disease. This could be especially useful for individuals with atypical symptoms or who are difficult to diagnose using traditional methods. Moreover, the differences in viral taxa may have prognostic implications. Specific viral taxa may be found to be associated with more severe disease or worse outcomes, this could be used to identify individuals who are at higher risk for complications and who may require more aggressive treatment. Conversely, certain viral taxa are found to be associated with milder disease or better outcomes, this could help identify individuals who may not require as aggressive treatment. The differences in viral taxa may also have therapeutic implications, this could potentially be targeted with antiviral therapies, further research is needed to fully understand the nature of these differences and their implications for patient care.

In interpreting metagenomic findings in Crohn’s disease, it is crucial to consider various factors that can potentially influence the gut microbiome, including ethnicity, sex, age of onset, lifestyle, known mutational signatures, and methodology (such as sequencing or antibody-based techniques). These factors may contribute to the observed differences in gut microbiome composition among individuals with Crohn’s disease and healthy controls. However, further research is needed to fully understand the complex interactions between these factors and the enteric virome in the context of Crohn’s disease. Polyomaviruses are non-enveloped, dsDNA viruses that are commonly found in the mammals. Two members of the family that have been confirmed to be linked to human diseases are the JC virus and the BK virus. These viruses have been known to induce tumors in animal models (Korup et al., 2013). In this study, a polyomavirus with a high degree of identity to JCV was identified in the fecal sample of a patient with Crohn’s disease. Interestingly, this virus was found to have a high positive rate (43.2%) in the disease group, but a low rate (3.2%) in the normal group. This finding suggests that the virus could be linked to the development of CD.

In a study published in 1999, the viral copy number of JCV was found to be significantly higher in colorectal cancer tissue than in normal colon tissue (Burnett-Hartman et al., 2008). It’s not hard to guess that the JC virus is also linked to inflammatory bowel disease, given that IBDs have a tendency to develop into tumors. While it is worth noting that patients with IBD are often treated with biological agents and immunomodulators, which have been shown to increase the risk of opportunistic infections (Dave et al., 2014). Studies have found that the use of these drugs is associated with the development of progressive multifocal leukoencephalopathy (PML; Van Assche et al., 2005), a disease thought to be related to JC virus infection. One study also found JC virus latency and urine viral shedding is frequent in immunosuppressed patients with Crohn’s disease (Verbeeck et al., 2008). However, another retrospective study reported the prevalence of CD patients exposed to JC virus is comparable with that of the general population (Bellaguarda et al., 2015). Therefore, JCV has been investigated as a possible link to CD, but the evidence for this association remains inconclusive. JC virus may be one of the pathogenic factors of CD, or it could be a chain of opportunistic infections. Further research is needed to elucidate the intricate mechanisms underlying this viral-host crosstalk, from the molecular pathways involved to the downstream effects on the gut ecosystem.

In this study, a novel anellovirus was detected in the feces sample of a patient with CD, adding to the growing body of research on the association between anelloviruses and various human diseases. Anelloviruses are commonly present in healthy human viromes, but elevated viral loads have been observed in patients with severe idiopathic inflammatory myopathies, cancer, and lupus (Hino and Miyata, 2007). A Bayesian phylogenetic tree constructed on the basis of the ORF1 of HuAV indicated that this novel anellovirus belonged to the group of *Alphatorquevirus* (TTV). In addition, anelloviruses from humans, gorillas, and chimpanzees clustered closely together, suggesting that there may be similarities in anellovirus tropism between these closely related hominin hosts. The rate of positive viral results was similar between the disease and the control groups (13.5 and 16.1%, respectively). These may indicate that the virus is widespread in humans, and not responsible for Crohn’s disease.

Numerous metagenomic investigations have revealed that the CRESS-DNA virus boasts a substantial diversity, much of which is unclassified, uncultured (Kazlauskas et al., 2018), and predominantly identified on the basis of conserved rolling circle replication proteins (Guo et al., 2018). In this study, a novel CRESS-DNA virus was detected in the fecal samples from a patient with CD. Phylogenetic analysis performed based on the amino acid sequences of Rep showed that the HuCV may represent a new unclassified CRESS-DNA virus. Despite differences in the virus-positive rates between the disease and control cohorts, the viruses that were most closely related to HuCV were extracted from aquatic organisms, indicating that the HuCV might not be a human virus but could have originated from the remnants of abalone, haddock, or other aquatic tissue products present within the digestive tract. Further research is necessary to probe the pathogenicity of the virus.

This article provides insight into the mysterious world of gut virome in CD patients, employing the cutting-edge tool of metagenomic analysis. The study sheds light on the prevalence of three viruses in the gut microbiota, namely a polyomavirus, an anellovirus, and a CRESS-DNA virus. However, this is only the tip of the iceberg, as much larger sample sizes and concrete evidence of viral replication are imperative to unravel the intricate associations between these gut viruses and the enigmatic world of intestinal disease. The future of gut virome research is as tantalizing as it is unpredictable, with new discoveries and challenges lurking around every corner.

## Data availability statement

The datasets presented in this study can be found in online repositories. The names of the repository/repositories and accession number(s) can be found in the article/Supplementary material.

## Ethics statement

The studies involving human participants were reviewed and approved by all individuals signed a written consent form prior to participation in the study, which was approved by the Medical Ethics Committee of the Affiliated Hospital of Jiangsu University (reference number:2020ahujs219). The patients/participants provided their written informed consent to participate in this study.

## Author contributions

WZ and XM investigated, conceived, and designed this study. YD, MW, and ZL collected the samples and data. YD performed the experiments and wrote the manuscript. MW analyzed the data. WZ and MX revised and edited the manuscript.

## Funding

This study was supported by the National Key Research and Development Programs of China (No. 2022YFC2603801) to and Funding for Kunlun Talented People of Qinghai Province, High-end Innovation and Entrepreneurship talents – Leading Talents (No. 202208170046), National Natural Science Foundation of China (Nos. 82072754 and 32170910), and Jiangsu Provincial Key Research and Development Program (No. BE2018689). All authors contributed to the article and approved the submitted version.

## Conflict of interest

The authors declare that the research was conducted in the absence of any commercial or financial relationships that could be construed as a potential conflict of interest.

## Publisher’s note

All claims expressed in this article are solely those of the authors and do not necessarily represent those of their affiliated organizations, or those of the publisher, the editors and the reviewers. Any product that may be evaluated in this article, or claim that may be made by its manufacturer, is not guaranteed or endorsed by the publisher.
