# Supplementary material for: Comparison of the gut virus communities between patients with Crohn’s disease and healthy individuals
Source: Front Microbiol. 2023 Jun 15;14:1190172. doi: 10.3389/fmicb.2023.1190172 (PMC10311494; doi:10.3389/fmicb.2023.1190172)

**Supplementary Data**

**Comparison of the gut virus communities between patients with Crohn’s Disease and healthy individuals**

Yuzhu Ding^a^, Mengtian Wan^a^, Zheng li^a^, Xiao Ma^c*^, Wen Zhang^b*^, Min Xu^a*^

*^a^ Gastroenterology department, the Affiliated Hospital of Jiangsu University, Zhenjiang,*

*Jiangsu 212001, China.*

*^b^ School of Medicine,* *Jiangsu University, Zhenjiang, 212013, China*

^c^ *Qinghai Institute of Endemic Disease Prevention and Control, Xining, Qinghai, China.*

^*^ Corresponding authors.

*E-mail addresses*: [maxiao0971@163.com](mailto:maxiao0971@163.com) (X.Ma) [z0216wen@yahoo.com](mailto:z0216wen@yahoo.com) (W. Zhang), peterxu1974@163.com (M. Xu).

**Supplementary Table S1** The summary of library information of CD patients

| Library ID | Sample type | Healthy status | No. of viral reads with E value < 10^−5^ | Library ID | Sample type | Healthy status | No. of viral reads with E value < 10^−5^ |
| --- | --- | --- | --- | --- | --- | --- | --- |
| 1 | Feces | CD | 3708 | 21 | Feces | CD | 2347 |
| 2 | Feces | CD | 286483 | 22 | Feces | CD | 3921 |
| 3 | Feces | CD | 151726 | 23 | Feces | CD | 201448 |
| 4 | Feces | CD | 6496 | 24 | Feces | CD | 27440 |
| 5 | Feces | CD | 11538 | 25 | Feces | CD | 3406 |
| 6 | Feces | CD | 3141 | 26 | Feces | CD | 12439 |
| 7 | Feces | CD | 438267 | 27 | Feces | CD | 10336 |
| 8 | Feces | CD | 350625 | 28 | Feces | CD | 1720 |
| 9 | Feces | CD | 269349 | 29 | Feces | CD | 23816 |
| 11 | Feces | CD | 638499 | 30 | Feces | CD | 13507 |
| 12 | Feces | CD | 311675 | 31 | Feces | CD | 396 |
| 13 | Feces | CD | 3373 | 32 | Feces | CD | 1682 |
| 14 | Feces | CD | 18153 | 33 | Feces | CD | 1016 |
| 15 | Feces | CD | 90635 | 34 | Feces | CD | 52352 |
| 16 | Feces | CD | 54791 | 35 | Feces | CD | 6948 |
| 17 | Feces | CD | 72693 | 36 | Feces | CD | 10336 |
| 18 | Feces | CD | 92285 | 37 | Feces | CD | 12439 |
| 19 | Feces | CD | 106142 |  |  |  |  |
| 20 | Feces | CD | 39661 |  |  |  |  |

**Supplementary Table S2** The summary of library information of healthy individuals

| Library ID | Sample type | Healthy status | No. of viral reads with E value < 10^−5^ |
| --- | --- | --- | --- |
| 38 | Feces | Heathy | 556655 |
| 39 | Feces | Heathy | 62181 |
| 40 | Feces | Heathy | 422844 |
| 41 | Feces | Heathy | 6610 |
| 42 | Feces | Heathy | 2477 |
| 43 | Feces | Heathy | 126 |
| 44 | Feces | Heathy | 109 |
| 45 | Feces | Heathy | 20 |
| 46 | Feces | Heathy | 645 |

**Supplementary Table S3** The demographic data and clinical characteristic of the patients and healthy individual included in this study.

| Library ID | Gender | Age | Blood Pressure | BMI | Healthy Status | Type of diagnosis |
| --- | --- | --- | --- | --- | --- | --- |
| 1 | male | 30 | 135/78 | 23.5 | Crohn’s Disease | ileocolitis |
| 2 | male | 36 | 123/82 | 22.2 | Crohn’s Disease | ileocolitis |
| 3 | female | 45 | 134/68 | 20.9 | Crohn’s Disease | ileitis |
| 4 | male | 30 | 121/64 | 19.9 | Crohn’s Disease | ileitis |
| 5 | male | 39 | 102/64 | 20.1 | Crohn’s Disease | ileocolitis |
| 6 | female | 24 | 135/89 | 22.2 | Crohn’s Disease | ileocolitis |
| 7 | female | 40 | 122/76 | 19.5 | Crohn’s Disease | ileitis |
| 8 | female | 30 | 108/63 | 23.5 | Crohn’s Disease | ileitis |
| 9 | female | 27 | 106/56 | 19.5 | Crohn’s Disease | ileitis |
| 10 | male | 41 | 140/88 | 19.5 | Crohn’s Disease | ileocolitis |
| 11 | female | 39 | 125/74 | 18.4 | Crohn’s Disease | ileitis |
| 12 | male | 30 | 123/65 | 23.2 | Crohn’s Disease | ileocolitis |
| 13 | male | 25 | 124/67 | 19.4 | Crohn’s Disease | ileocolitis |
| 14 | male | 37 | 123/69 | 22.1 | Crohn’s Disease | ileitis |
| 15 | male | 32 | 106/57 | 21.1 | Crohn’s Disease | ileitis |
| 16 | female | 41 | 112/70 | 22.4 | Crohn’s Disease | ileocolitis |
| 17 | female | 44 | 105/63 | 19.1 | Crohn’s Disease | ileocolitis |
| 18 | female | 37 | 112/65 | 19.5 | Crohn’s Disease | ileocolitis |
| 19 | male | 25 | 102/59 | 19.6 | Crohn’s Disease | ileocolitis |
| 20 | male | 33 | 119/71 | 22.2 | Crohn’s Disease | ileocolitis |
| 21 | female | 40 | 125/74 | 18.4 | Crohn’s Disease | ileitis |
| 22 | female | 40 | 103/68 | 20.3 | Crohn’s Disease | ileitis |
| 23 | male | 34 | 116/84 | 20.5 | Crohn’s Disease | ileocolitis |
| 24 | male | 48 | 119/71 | 22.0 | Crohn’s Disease | ileocolitis |
| 25 | male | 21 | 120/65 | 23.1 | Crohn’s Disease | ileocolitis |
| 26 | female | 33 | 118/83 | 22.5 | Crohn’s Disease | ileocolitis |
| 27 | female | 39 | 113/63 | 20.4 | Crohn’s Disease | ileitis |
| 28 | male | 29 | 118/76 | 19.1 | Crohn’s Disease | ileitis |
| 29 | female | 40 | 123/80 | 20.4 | Crohn’s Disease | ileitis |
| 30 | male | 29 | 103/63 | 19.3 | Crohn’s Disease | ileitis |
| 31 | male | 23 | 124/78 | 19.7 | Crohn’s Disease | ileitis |
| 32 | female | 26 | 95/62 | 18.4 | Crohn’s Disease | ileocolitis |
| 33 | female | 23 | 102/63 | 23.1 | Crohn’s Disease | ileocolitis |
| 34 | female | 31 | 109/64 | 19.3 | Crohn’s Disease | ileocolitis |
| 35 | male | 33 | 123/70 | 23.8 | Crohn’s Disease | ileitis |
| 36 | male | 26 | 121/67 | 22.4 | Crohn’s Disease | ileitis |
| 37 | female | 47 | 106/61 | 21.6 | Crohn’s Disease | ileocolitis |
| 38 | female | 36 | 132/70 | 20.2 | Healthy group | - |
| 39 | female | 40 | 129/62 | 19.6 | Healthy group | - |
| 40 | male | 25 | 110/61 | 22.4 | Healthy group | - |
| 41 | female | 45 | 125/82 | 18.9 | Healthy group | - |

**Continuation table S3**

| 42 | male | 47 | 127/83 | 20.5 | Healthy group | - |
| --- | --- | --- | --- | --- | --- | --- |
| 43 | male | 34 | 143/97 | 22.1 | Healthy group | - |
| 44 | female | 24 | 92/64 | 18.1 | Healthy group | - |
| 45 | male | 23 | 117/86 | 21.7 | Healthy group | - |
| 46 | male | 42 | 121/84 | 21.0 | Healthy group | - |

**Supplementary Fig. S1** Positive screening results of HuPyV in all 68 samples. Fecal samples collected from patients with Crohn’s disease are labeled in red.


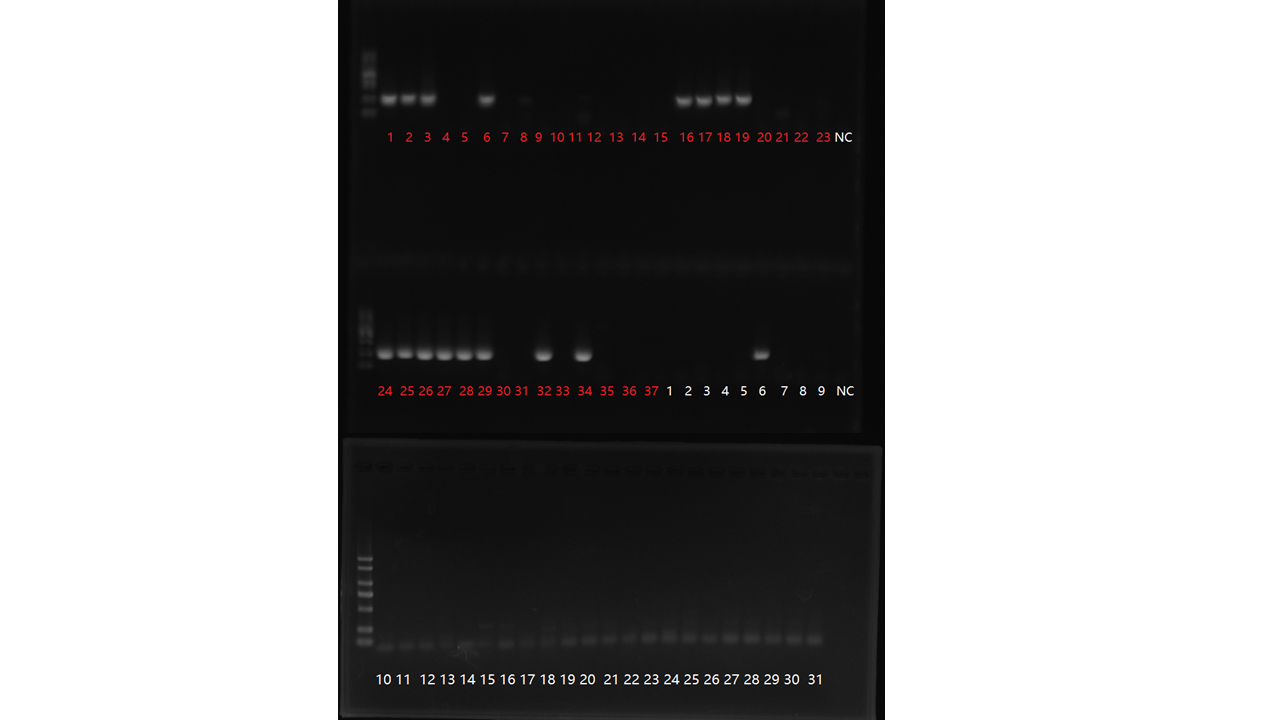


**Supplementary Fig. S2** Positive screening results of HuAV in all 68 samples. Fecal samples collected from patients with Crohn’s disease are labeled in red.


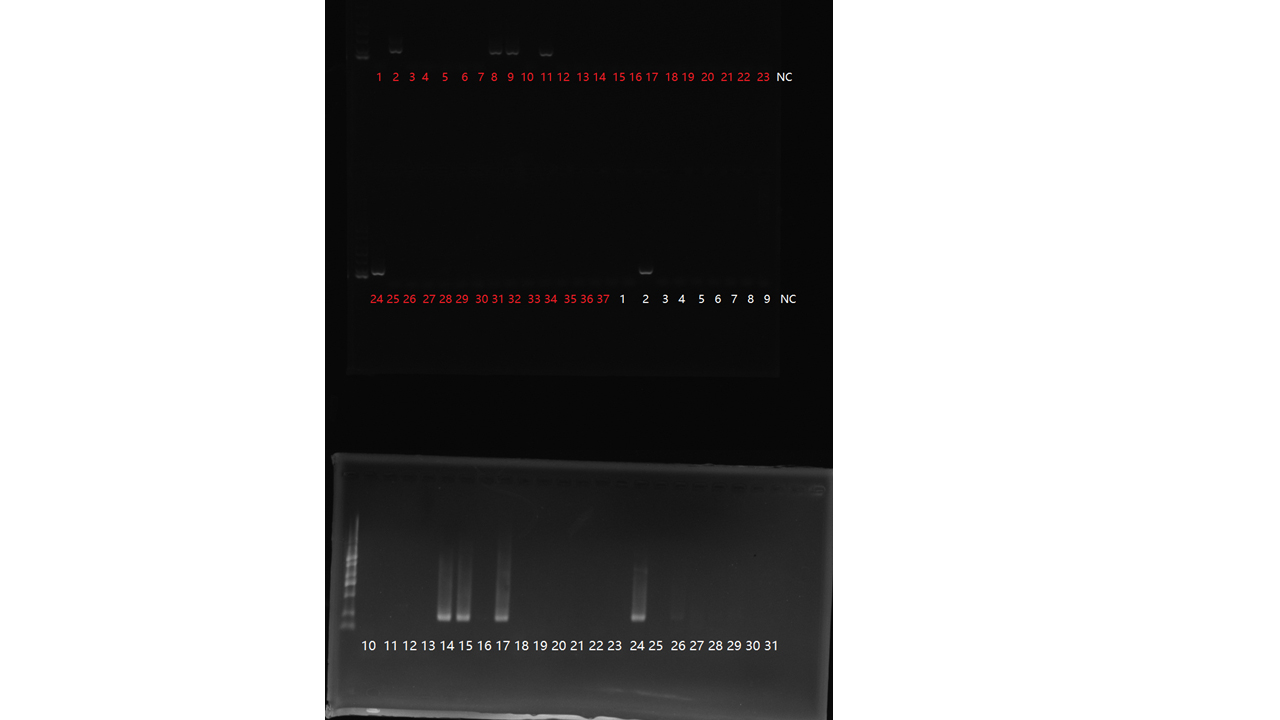


**Supplementary Fig. S3** Positive screening results of HuCV in all 68 samples. Fecal samples collected from patients with Crohn’s disease are labeled in red.


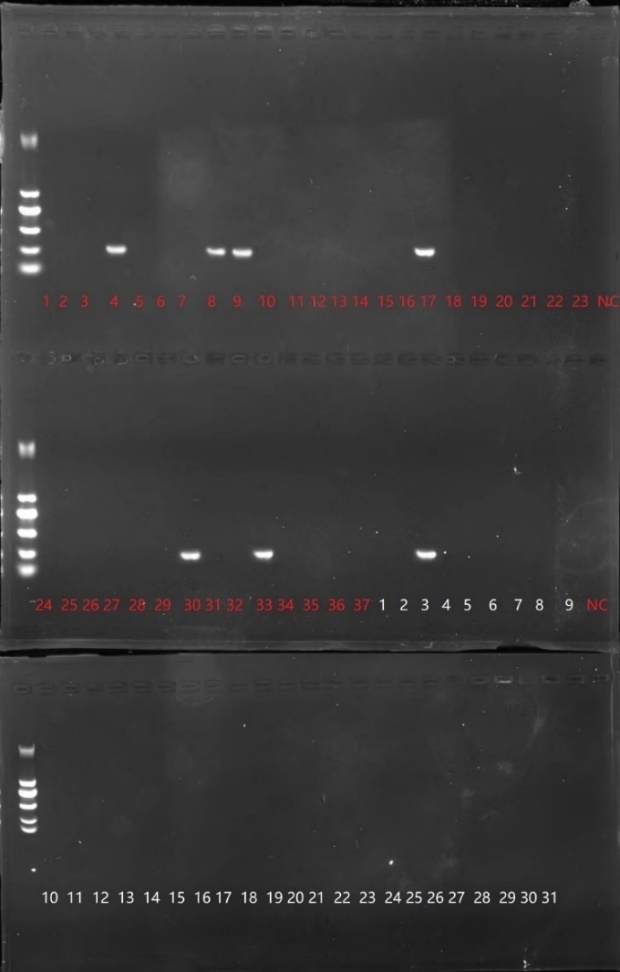

Supplement: Supplementary file 1 [file Data_Sheet_1.docx]
